# Effect of aeration, iron and arsenic concentrations, and groundwater matrix on arsenic removal using laboratory sand filtration

**DOI:** 10.1007/s10653-020-00671-7

**Published:** 2020-07-21

**Authors:** Cynthia A. Coles, Danial Rohail

**Affiliations:** 1 Department of Civil Engineering, Memorial University of Newfoundland, 40 Arctic Avenue, St. John’s, NL A1B 3X7 Canada; 2 Infrastructure Ontario, Suite 2000, 1 Dundas St. West, Toronto, ON M5G 1Z3 Canada

**Keywords:** Arsenic removal, Iron–arsenic ratio, Sand filtration, Groundwater matrix, Metals

## Abstract

Natural groundwater from the towns of Wabana and Freshwater and treated well water from the town of Wabana in Newfoundland and Labrador, Canada were tested separately and together in sand columns to study the removal of arsenic. The most ideal conditions for arsenic removal appeared to include an arsenic concentration of approximately 35 µg/L and lower, an Fe:As mass ratio in the order of 65 and lower, and aeration of the sand media. Active aeration by pumping air though the filter, passive aeration by scraping off top layers of sand and virtual aeration by diluting the strength of the water being treated, were employed and compared. For tests where groundwater from the towns of Wabana and Freshwater was combined, arsenic removal was optimized and other elements, in addition to iron, were also correlated with effluent arsenic. Further, for these same tests there was a gradual increase in effluent pH that could have been due to oxygen depletion or gradually more reducing conditions in the sand column. Where Ni, Mn and Zn were correlated with effluent arsenic it was concluded that the increase in pH increased heavy metal removal and arsenic release. In the test where the treated Wabana water made up a greater proportion of the mix than the Wabana groundwater, lithium was also correlated with arsenic.

## Introduction

Many soils in Newfoundland and Labrador (NL), Canada have elevated levels of arsenic and so arsenic in drinking water from wells in the rural communities is not uncommon (Rageh 2008).

Up to 3000 mg/kg of arsenic was measured in the iron sulfide sedimentary rock on Bell Island in NL (Onishi and Sandell 1955) and arsenic is often found in sulfide minerals and in sedimentary rock that is high in iron, manganese and aluminum (Jovanovic et al. 2011; Onishi and Sandell 1955). The average abundance of arsenic in the earth’s crust in the USA is about 7.2 mg/kg (Schacklette and Boerngen 1984) and in uncontaminated groundwater the arsenic can be less than 1–2 μg/L (Jovanovic et al. 2011).

Arsenic has four oxidations states (− III, 0, + III and + V), though arsenite or As(III) and arsenate or As(V) are most important in soils and water. Arsenite dominates under anoxic/reducing conditions and arsenate is more abundant under aerobic/oxidizing conditions (Sarkar and Paul 2016). Groundwater can contain either or both species (Jovanovic et al. 2011) and each species can be present under either oxidizing or reducing conditions as they tend to be stable and slow to change state (Shaw 2006). Arsenite can be converted into arsenate under oxidizing conditions and in the presence of sunlight (García et al. 2004) and arsenate could be the predominant species in groundwater in NL (Rageh 2008).

Well water is the main source of drinking water in rural NL and arsenic concentrations are typically higher in groundwater than in surface water (Wang and Mulligan 2006). Weathering of arsenic containing minerals in aquifers leads to arsenic in groundwater and perhaps most commonly by oxidation of the minerals (Sarkar and Paul 2016). Near to Los Ralos (Tucumán, Argentina) 2 months of dry weather conditions depressed the water table and decreased the opportunity for mineral weathering and the concentration of arsenic in the groundwater (García et al. 2004). Conversely, the rainy NL climate and the province’s close proximity to salt water along the coast can promote more weathering of arsenic into groundwater (Rageh et al. 2007).

Adsorption, filtration, and coagulation or co-precipitation with iron(III) hydroxide, aluminum sulfate and manganese salts are commonly used to remove arsenic for drinking water purposes and point-of-use devices use a combination of techniques (Rageh 2008). Usually the goal is to achieve at least the WHO standard of 10 μg/L of arsenic in treated water though more stringent guidelines would be preferable (Wang and Mulligan 2006; Bordoloi et al. 2013). Obtaining less than 10 μg/L of arsenic is possible with ion exchange, activated alumina and reverse osmosis but the technology cannot be afforded by everyone (Berg et al. 2006; Gu et al. 2005) and the sustainability of the methods has been questioned. For example, removing arsenite by nanofiltration or reverse osmosis is energy intensive because first arsenite is usually oxidized to arsenate, and after membrane treatment the beneficial minerals removed need to be replenished, leading to the complete process being expensive and complex (Bolisetty et al. 2019).

The U.S. Environmental Protection Agency (U.S. EPA) considers arsenic a priority pollutant and follows the WHO standard for drinking water of 10 μg/L (Jovanović et al. 2011; Sazakli et al. 2015). Arsenic is carcinogenic and highly toxic, especially the inorganic forms in water or As(III) and As(V) and most arsenic compounds are odorless and tasteless and so might go undetected. Chronic exposure can lead to various illnesses, including but not limited to, skin, lung, bladder and kidney cancers, anemia, leucopenia, and neurological and cardiovascular disorders (Wang and Mulligan 2009; Jovanović et al. 2011).

When Berg et al. (2006) used sand filtration on groundwater taken from the Red River Delta in Vietnam they observed that arsenic removal increased as the proportion of iron to arsenic increased. When the molar ratio of Fe to As was ≥ 250 they could remove arsenic to < 10 μg/L and when the molar Fe/As ratio was ≥ 50 they could achieve arsenic removal to < 50 μg/L (Berg et al. 2006).

The oxidation of ferrous iron (Fe^2+^) to ferric (hydr)oxide (Fe^3+^) precipitates in groundwater also assists the oxidation of arsenite to arsenate, and can lead to excellent removal by sand filtration of the iron oxides which have a strong affinity for and tend to become bonded with the arsenate (Roberts et al. 2004). Silicate or phosphate in groundwater can also inhibit arsenic removal by the iron oxides, though Ca^2+^ and Mg^2+^ can lessen the negative effect of Si on arsenate uptake (Roberts et al. 2004).

The success of any system for removal of arsenic is influenced by the water properties (Sazakli et al. 2015) and the groundwater composition would be important in a sand filter system. For example, in Bangladesh where arsenite is the dominant species in groundwater and the ferrous iron concentration is low, passive removal of arsenic without iron supplementation could be ineffective (Roberts et al. 2004). When arsenite is the dominant species it is usually oxidized to arsenate because the latter is easier to remove (Roberts et al. 2004).

The objective of this research was to obtain a better understanding of how the test conditions such as the type of aeration, the properties of the sand filter media, and the properties of the groundwater and treated well water, tested separately and in combination, could improve arsenic removal. This was accomplished by using laboratory column tests with sand as the filter media. Given that groundwater can vary considerably based on the geographic location it is useful to test actual groundwater from more locations to study arsenic removal.

## Materials and methods

Arsenic, iron and other elements in water samples were measured using a Hewlett-Packard 4500 series Inductively Coupled Plasma Mass Spectrometer (ICP-MS) in the TERRA-ICP-MS facility at Memorial University of Newfoundland (MUN) during 29 analytical sessions in 2011 and 2012. All water samples were passed through a 0.45 μm filter paper and acidified with HNO_3_ before analysis.

The detection limit (DL) when using ICP-MS will vary with the session, with the blank being used and with the tuning of the instrument and could be affected by such things as temperature and humidity (John Allen and Inês Nobre Silva, TERRA-ICP-MS facility, MUN, personal communications, July 2018 and May 2020). The mean value of the DL for each relevant element was: As 0.95 μg/L, Mn 1.2 μg/L, Li 2.4 μg/L, Al 11 μg/L, Br 15 μg/L, Zn 16 μg/L, Fe 160 μg/L, Ni 210 μg/L, Cl 4.8 mg/L, and S 41 mg/L. The standard deviation among the DL values for each element was: As 1.66 μg/L, Mn 2.57 μg/L, Li 4.66 μg/L, Al 26.0 μg/L, Br 22.7 μg/L, Zn 53.3 μg/L, Fe 215 μg/L, Ni 950 μg/L, Cl 14.3 mg/L and S 110 mg/L.

To overcome species interferences during each ICP-MS session, known standard multi-element solutions as indicated by Friel et al. (1990) were run to calibrate the instrument, and mono-element solutions (of Sc, Re, Rh and Th) were measured to control instrument drift. The ICP-MS took multiple readings for each sample and recorded an average value (Inês Nobre Silva, TERRA-ICP-MS facility, MUN, personal communication, May 2020). The advantage of using ICP-MS is its ability to measure multiple elements at one time.

The sand, supplied by Capital Ready Mix in St. John’s, was concrete sand from Black Mountain that had been surface mined and washed and screened to remove particles smaller than 0.08 mm and 99.8% of particles were less than 5 mm in size. The sand had a bulk density of 1.47 g/cm^3^, a porosity of 34.2%, and a uniformity coefficient of > 7.5. (M. Lynch, AMEC Earth & Environment, 10 June 2011).

**Fig. 1:**
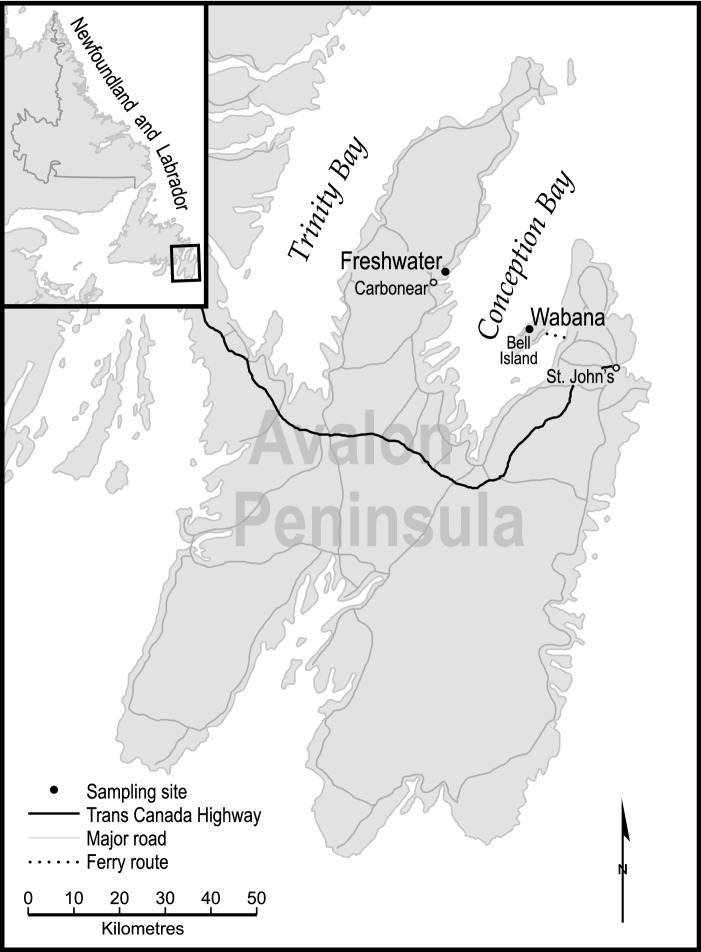
The sampling sites are at Wabana and Freshwater on the Avalon Peninsula, Eastern Newfoundland

Groundwater samples were collected from the towns of Wabana and Freshwater and treated well water was collected from the Wabana distribution network. These two towns are less than 75 min by road from St. John’s, the provincial capital and the three locations are highlighted on the map in Fig. 1.

The groundwater collected from Wabana is referred to as the high strength Wabana groundwater or the Wabana groundwater and had iron and arsenic concentrations of 11,437 μg/L and 62.7 μg/L, respectively. Treated well water from Wabana’s distribution network is referred to as the treated Wabana water (and contained 86.3 μg/L of iron and 4.5 μg/L of arsenic). Column tests with the high strength Wabana groundwater and with mixtures of the high strength and treated Wabana waters in ratios of 1:1 and 1:3 were conducted to examine the impact of dilution on arsenic removal by the sand filter media. For the tests with the two Wabana waters the influent water was transferred into 1 L bottles, filled to zero airspace and sealed in an effort to slow the oxidation of Fe^2+^ to Fe^3+^.

Groundwater collected from the town of Freshwater had arsenic and iron concentrations were 29.7 μg/L and below the instrument detection limit, respectively. In the last column tests, high strength Wabana groundwater was combined with groundwater from the town of Freshwater in 1:10, 2:10 and 3:10 ratios, respectively, referred to as Mix1, Mix2 and Mix3, to see if the iron in the high strength Wabana groundwater would compensate for the iron deficient groundwater from the town of Freshwater in order to improve arsenic removal in the sand columns.

For the final tests that combined groundwater from Wabana and Freshwater it was necessary to return to the two towns to collect more water and there was some variability in the element concentrations compared to the first collection, but weather and seasonal conditions before sampling could cause this (García et al. 2004). All groundwater samples tested in the sand columns were supplied in their natural states without any pH or other adjustment, other than creating the abovementioned mixtures where indicated.

**Fig. 2:**
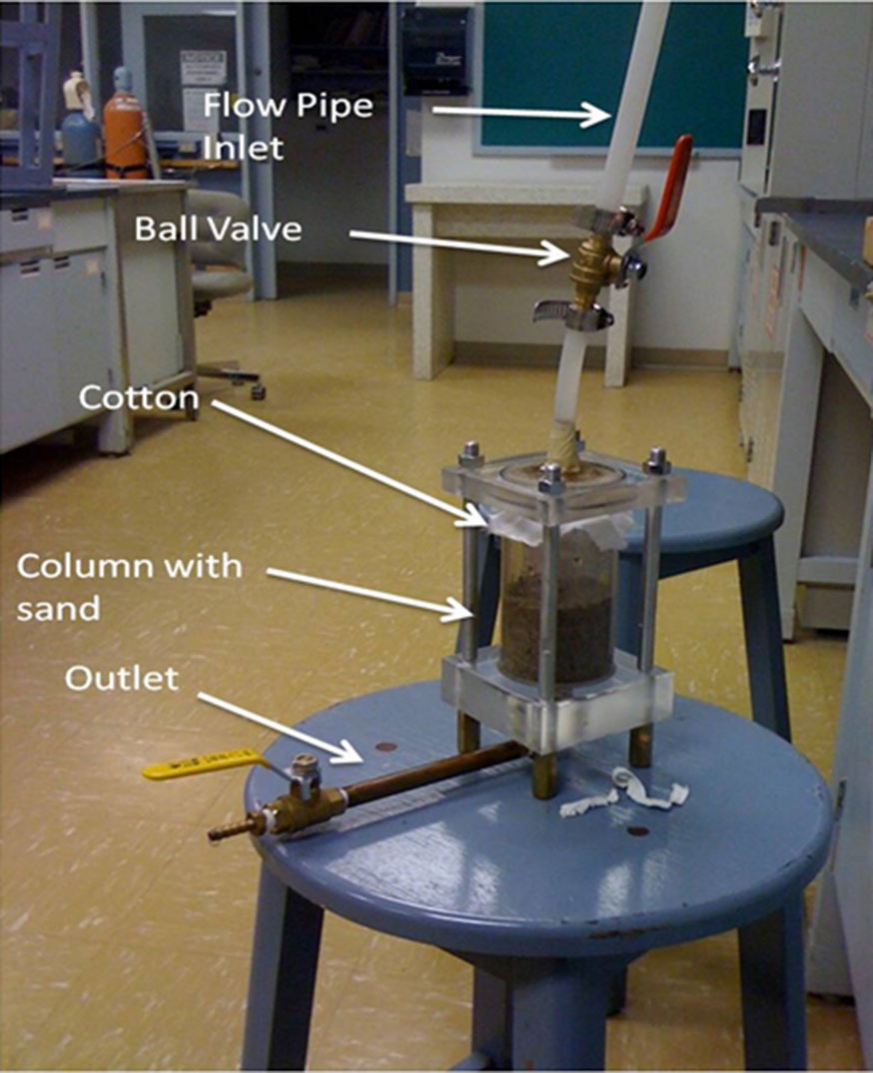
The column used in the lab tests

For all the column tests, the sand was placed in a vertical cell (shown in Fig. 2) with one thickness of cloth (55% cotton, 45% polyester, 180 thread count) at the top inlet to distribute the water and three thicknesses of cloth at the bottom outlet to prevent migration of fines. (A blank test with the cloth alone showed that arsenic removal by the cloth was negligible.) The column internal diameter was 6.7 cm and the 14 cm high column was filled to a height of 6.7 cm (giving a sand volume of 236 cm^3^ and one pore volume of 80.7 cm^3^). Upper and bottom cell plates were fitted with O-rings to ensure water tightness and the cells were fabricated by Technical Services (at MUN) of an acrylic material purchased from E M Plastics.

The sand was washed in the lab in 60 °C distilled water, allowed to drain with some fines removal, and dried in an oven at 105 °C for 24 h. Mauclaire et al. (2006) operated a slow sand filter (SSF) with sand particles ranging in size from 0.2 to 2.0 mm compared to approximately 0.1–5.0 mm in this study. A slow sand filter (SSF) contains sand grains that are smaller in size than those used in a rapid sand filter (RSF) and a less uniform size is the norm since removal is by surface (versus depth) filtration and filter renewal is accomplished by scraping off surface layers of sand for washing and reuse (versus backwashing) (Crittenden and Montgomery Watson Harza 2005).

Before the start of each column test 1 L of distilled water was passed through the cell until the water ran clear and had removed any fines that could pass through the cloth. A 1 L aspirator bottle containing the source water, at a height of 1.2 m above the column inlet (unless otherwise stated) enabled gravity flow through the column.

For the tests that employed aeration, a non-lubricated Gast vacuum pump was used to draw air through the sand media to oxidize ferrous iron in the groundwater. The pump could generate 64.8 cm-Hg of vacuum at a pressure of 414 kPa and had a maximum output of 53.8 L/min.

For the Mix1, Mix2 and Mix3 column tests a Masterflex variable speed drive peristaltic pump (6–600 rpm) with a variable flow rate (3.6 × 10^−4^ to 3.4 L/min) was employed to increase the filtration rate. In these three column tests, the water was passed at a flow rate of approximately 2.1 × 10^−3^ L/min (or 0.125 L/h).

## Results and discussion

### High strength Wabana groundwater column tests and aeration effects

Three column tests were conducted with only the high strength Wabana groundwater. In the first of these tests no effort was made to improve the supply of air to the system. In the second test, 1 cm from the top surface of the sand was scraped off after 1.6 L and after 1.8 L of effluent had been collected from the outlet of the column, making the treatment similar to the operation of a SSF. In the third test, the sand column was aerated by sucking air with the vacuum pump for 2 h after approximately every 0.5 L of water was passed through the column. Though conditions in the third test were not the same as in a RSF, there were similarities in that the process was more energy intensive than in a SSF and there was the possibility that a greater depth of the sand media was used.

Mohapatra et al. (2002) describe the Leiduin plant supplying Amsterdam with 70 million m^3^ of treated surface water per year, as incorporating both a RSF early in the treatment train and a SSF as the finishing or polishing step. In a plant upgrade to increase the capacity by 13 million m^3^/year, they also proposed a SSF immediately prior to the final reverse osmosis treatment. Their study concluded that each sand filtration process had a negligible environmental impact.

Where there was a strong correlation between effluent concentrations of arsenic and iron, the correlation coefficient, r (calculated with Microsoft Excel) and the level of confidence (LOC) or probability of the correlation occurring, are shown. The LOC was estimated by comparing the value of r with the critical values given in Table A2 of “Statistics in Kinesiology” (Vincent and Weir 2012). Adsorbents containing iron are effective for removing arsenic (Gu et al. 2005; Berg et al. 2006; Meng et al. 2002) and the high concentration of iron in the groundwater could have assisted the uptake of arsenic in these tests.

In the first test in Table 1, the acceptable concentration for arsenic in drinking water of 10 μg/L was exceeded when between 1.6 and 2 L of water had passed through the column. During this test deposition of iron on the surface of the sand appeared to be blocking the pores and it was thought this could have limited the iron oxidation and adsorption of arsenic deeper within the sand. The permeability of iron-based filters can decrease over time due to oxidation of iron and this has led to the recent development of more porous iron-containing filters (Smith et al. 2017).

**Table 1:** Influent (0.0 L) and effluent concentrations of As and Fe in the column tests for the high strength Wabana groundwater only

No extra air supply to the sand column			Top sand surface scraped off after 1.6 L and 1.8 L			2 h aeration after every 0.5 L of water was passed		
Volume (L)	As (μg/L)	Fe (μg/L)	Volume (L)	As (μg/L)	Fe (μg/L)	Volume (L)	As (μg/L)	Fe (μg/L)
0.0	62.7	11,437	0.0	62.7	11,437	0.0	62.7	11,437
0.2	0.607	47.01	1.6	8.51	2076	1.4	0.50	DL^b^
1.4^a^	6.35	1385	1.8	9.63	1691	2.4	1.69	DL^b^
1.6	8.51	2076	2.0	9.62	1684	3.0	5.14	598
2.0	19.73	4414				3.5	10.68	1588
						4.5	26.58	4179
r		0.998				r		0.973
LOC		> 99%				LOC		≈ 97.5%

^a^This measurement only is the average of duplicate tests

^b^DL means value was below the instrument detection

To overcome the iron deposition of the sand in the first test, 1 cm of the top surface of the sand was removed after 1.6 L and 1.8 L of effluent were collected, for the second test. Exposing the underlying sand and pores to the air appeared to contribute to greater oxidation of iron and uptake of the arsenic. After the collection of 2.0 L of effluent, the arsenic and iron concentrations were only 9.62 and 1684 μg/L, respectively, compared to 19.73 and 4414 μg/L for the first test.

The third test with regular aeration of the sand column resulted in the best removal of arsenic and iron because between 3.0 and 3.5 L of water could be passed before the effluent arsenic concentration exceeded 10 μg/L.

In general, aeration can help convert Fe^2+^ ions into Fe^3+^ ions and oxidize arsenite into arsenate which is better removed by ferric oxides (Roberts et al. 2004). Mixtures of ferric oxyhydroxide (FeOOH) and manganese dioxide (MnO_2_) can both enhance arsenic removal and both are able to oxidize arsenite into arsenate and adsorb arsenate (Ocinsaki et al. 2016) although in these three tests no significant correlation was observed between arsenic and manganese.

Shaw (2006) conducted sequential extraction experiments on sediments where the mass of Mn was less than 3% of the mass of the Fe but where a higher proportion of Mn, estimated at 82% of the total Mn and present as manganese oxides (represented by the extractable phase), appeared to be bonded with arsenic. In this study, the mass of Mn in the high strength Wabana groundwater was about 25% of the mass of Fe (2819 μg/L compared to 11,437 μg/L).

Zhang et al. (2014) concluded that a binary Fe–Mn oxide mineral of the form FeOOH-MnO_x_ (1.5 < *x* < 2) was better at removing arsenic (both as arsenite and arsenate) at neutral pH than either FeOOH or MnO_x_ separately. They proposed that mostly the MnO_x_ component of the binary oxide converted arsenite to arsenate and then the FeOOH component adsorbed the arsenate. It is possible that arsenite was not abundant in these tests as no positive correlation between effluent arsenic and Mn was observed.

During some ICP-MS runs the iron concentrations and to a lesser extent the arsenic concentrations in the water samples were less than the instrument detection limits and this is indicated by DL or alternatively the data for iron and arsenic were omitted if many other effluent measurements were available.

A problem in the third test during aeration of the sand with the vacuum pump was that the sand became compressed and some of the smaller grains of sand could have been forced into the larger pores within the sand media. This could also have reduced the potential of the media to remove arsenic. Typically, the smallest grain size in slow sand filters is about 0.2 mm while in this study some sand grains that may have been as small as 0.1 mm. For a sand that is not selectively graded there is the possibility of smaller grains filling the voids formed by larger particles (Davis 2011).

### Column tests on two Wabana waters combined

Column tests four and five were conducted with high strength Wabana groundwater and the treated Wabana water in 1:1 and 1:3 ratios, respectively. Since the two new waters formed were more dilute in iron and arsenic than the high strength Wabana groundwater, the naturally occurring oxygen content relative to the iron and arsenic was proportionately greater. In these two tests when the arsenic in the effluent approached approximately 7.0 μg/L, the sand was aerated with the vacuum pump for 1 h and after every additional 0.5 L of water was passed. Table 2 shows that the aeration reduced the arsenic in the effluents and the tests were continued until the arsenic concentrations again increased. The r and LOC values in Table 2 were obtained using the data from both before and after aeration.

**Table 2:** Concentrations of As and Fe for the 1:1 and 1:3 ratios of Wabana waters and with aeration starting after the arsenic in the effluent approached 7.0 μg/L

1:1 ratio of high strength to treated Wabana waters			1:3 ratio of high strength to treated Wabana waters			
Volume (L)	As (μg/L)	Fe (μg/L)	Volume (L)	As (μg/L)	Fe (μg/L)	Li (μg/L)
0.0	33.6	5722	0.0	19.0	2920	17.2
1.4	2.70	294	1.5	1.47	94.9	2.64
2.4	4.84	597	3.0	4.27	143	8.13
3.0	5.74	910	4.0	4.76	583	13.94
3.4	1.72	249	5.0	3.96	353	12.57
3.7	7.10	995	6.0	1.78	32.9	7.55
4.0	6.96	1134	6.5	6.98	419	16.9
Start of aeration			7.5	3.82	206	13.06
4.2	6.21	1184	8.5	3.00	235	9.45
5.0	4.35	602	9.5	5.70	454	14.42
5.5	1.65	56.9	10.5	6.23	606	15.76
6.0	1.36	171	Start of aeration			
8.0	7.08	463	14.0	2.51	392	1.91
			17.0	2.74	198	18.10
			18.0	5.16	808	15.43
r		0.848	r		0.698	0.703
LOC		> 99%	LOC		> 99%	> 99%

Iron was well correlated with the arsenic in the two tests, as was lithium in the 1:3 ratio test of the two waters although the absolute lithium concentration was low. There have been only a few studies of lithium in drinking water, and drinking water quality guidelines for lithium do not yet exist although there is growing awareness of potential health problems due to lithium exposure (Coincha et al. 2010; Onorato et al. 2017) likely due to the rise in use of lithium ion batteries in recent decades. Baysal and Gunduz (2016) found that the sources of arsenic and lithum in natural surface waters were geothermal discharges and groundwater intrusions. Lithium in groundwater would occur mostly as Li^+^ (Onorato et al. 2017). The authors have not seen any studies of arsenic and lithium in drinking water or groundwater where lithium could have been important to or correlated with arsenic removal.

The treated Wabana water had a greater lithium content than the high strength Wabana groundwater and the arsenic was more amenable to removal in the 1:3 ratio of Wabana waters that contained more of the treated Wabana water. So as not to exceed approximately 7.0 µg/L of arsenic in the effluent only 8.0 L of the 1:1 water but 18.0 L of the 1:3 water could be treated or 2.5 times as much water while the difference in the initial arsenic contents was less than 2 times. It is unknown if lithium played any role in assisting the removal of arsenic. The effect of dilution of arsenic in the water could have been important and is discussed further in the next section.

### Comparison of column tests that employed aeration

Table 3 compares information from the three column tests where active aeration was employed. The factors considered include the influent arsenic and iron concentrations, correlations between arsenic and iron in the effluent, initial iron to arsenic mass ratios, and the volume of water treated and total arsenic uptake by the sand before arsenic concentrations in the effluent approached approximately 7.0 μg/L.

**Table 3:** Comparison among tests that employed aeration with high strength Wabana groundwater alone and combined with treated Wabana water in 1:1 and 1:3 ratios

Ratio of high strength to treated Wabana waters	Influent As (μg/L)	Influent Fe (μg/L)	Influent Fe:As mass ratio	Correlation between effluent As and Fe (r)	Volume of water treated (L)	Arsenic uptake until 7 µg/L in effluent (μg)
1:0	62.7	11,437	182	0.973	3.0	80
1:1	33.6	5722	170	0.848	8.0	115
1:3	19.0	2920	154	0.698	18.0	153

Table 3 shows that the greater the Fe:As mass ratio, the greater the correlation between iron and arsenic in the effluent. In the column test with only the high strength Wabana groundwater, iron had been deposited on the top of the sand and this limited further arsenic removal. In the more dilute waters, there was greater total arsenic uptake and, relative to the As and the Fe, it is expected there was more oxygen naturally occurring to compliment the aeration and this could have provided more space for iron oxide formation and arsenic uptake.

Diluting the arsenic concentration in the water to give a greater oxygen to arsenic ratio could be seen as a form of passive aeration. It would be interesting to see if with colder water, naturally richer in oxygen, there could be greater arsenic uptake. Smith et al. (2017) concluded that reducing the flow rate through a filter with layers of sand, activated carbon and iron filings increased arsenic removal because there was greater contact time between the iron filings and water allowing for more iron oxides to form.

Berg et al. (2006) emphasized the importance of Fe:As ratio for sand filtering of arsenic and reported that greater Fe:As ratios produced clearer effluent from household sand filters. The Fe:As ratios in the tests considered in Table 3 are still high and they do appear to be a factor in the removal of arsenic. The difference here is that the total uptake of arsenic was slightly greater where the Fe:As ratio was lower but this could be due to the effect of the dilution of the arsenic in the water relative to the naturally occurring oxygen. Also because of the complexity of natural groundwater, it is difficult to make comparisons of groundwater from different locations.

### Column test on groundwater from the town of Freshwater

In the sixth column test with the groundwater from the town of Freshwater (Table 4), arsenic in the effluent exceeded 7 μg/L after only 1.0 L of water was passed compared to 1.4 L for the high strength Wabana groundwater (Table 1) despite that the Wabana groundwater contained double the arsenic of the groundwater from the town of Freshwater. Since high iron concentrations are effective for passive removal of arsenic (Roberts et al. 2004) and the groundwater from the town of Freshwater had only 5% of the iron of the high strength Wabana groundwater, the (relatively and absolutely) smaller iron content could have contributed to the smaller total arsenic removal and the smaller volume of water that could be treated. These results further suggest iron is important in the arsenic removal.

**Table 4:** Concentrations of As and Fe for the column test with groundwater from the town of Freshwater

Volume (L)	As (μg/L)	Fe (μg/L)
0.0	29.7	DL^a^
1.0	4.88	117
1.2	8.22	270

^a^DL means value was below the instrument detection limit

### Column tests on groundwater from the towns of Wabana and Freshwater combined

Column test results for mixtures of groundwater from the towns of Wabana and Freshwater are presented in Tables 5, 6 and 7 (where NA indicates a value that was not measured and is thus not available). The Mix1, Mix2 and Mix3 waters had 1:10, 2:10 and 3:10 ratios, respectively, of groundwater from the towns of Wabana and Freshwater and the tests included freshly collected samples of these two waters. Some variation in the concentrations of elements between the two sampling times was likely due to seasonal or monthly changes and the wetness or dryness of conditions preceding sampling (García et al. 2004). Correlations of arsenic with Fe and other elements are included whenever the LOC values were > 97.5, to match LOC values with iron in the preceding tests.

**Table 5:** Mix1 column test results with influent (0 h, 0 L) and effluent concentrations for As and elements correlated with As for LOCs > 97.5, and pHs as a function of time

Time (h)	Volume (L)	As (μg/L)	Cl (mg/L)	Br (μg/L)	Ni (μg/L)	Mn (μg/L)	S (mg/L)	pH
0	0	33.6	130	163	2.1	196	DL^a^	8.0
8	0.9	DL^a^	115	20.3	DL^a^	21.3	DL^a^	8.4
36	4.3	4.5	92.0	115	18.9	DL^a^	6.25	8.4
68	8.1	6.36	91.1	120	13.6	219	5.44	8.4
84	10	5.86	92.4	110	14.1	249	13.5	8.4
200	23.7	11.5	88.3	120	9.07	218	4.37	8.4
240	28.4	13.1	134	197	5.5	257	25.7	NA^b^
290	34.4	16.9	141	182	2.82	204	DL^a^	8.5
315	37.3	20.4	132	173	1.51	173	DL^a^	8.5
325	38.5	23.9	141	181	2.26	178	DL^a^	8.5
r			0.858	0.800	− 0.940	− 0.776	0.920	
LOC			> 99	> 99	> 99	> 97.5	> 97.5	

^a^DL means value was below the instrument detection limit

^b^NA means no measurement was taken

**Table 6:** Mix2 column test results with influent (0 h, 0 L) and effluent concentrations of As and Zn, and pHs as a function of time

Time (h)	Volume (L)	As (μg/L)	Zn (μg/L)	pH
0	0	34.3	120	7.9
8	1	DL^a^	2040	8.3
40	5	3.4	902	8.4
56	6.9	5.7	283	8.4
80	9.9	11.0	107	8.4
120	14.9	7.0	145	8.4
160	19.8	9.6	135	8.4
240	29.7	8.5	339	NA^b^
280	34.7	6.6	162	8.4
296	36.6	8.3	239	8.3
315	39	8.7	119	8.2
r			− 0.764	
LOC			> 97.5%	

^a^DL means value was below the instrument detection limit

^b^NA means no measurement was taken

**Table 7:** Mix3 column test results with influent (0 h, 0 L) and effluent concentrations of As and elements correlated with As for LOCs > 97.5, and pHs as a function of time

Time (h)	Volume (L)	As (μg/L)	Al (μg/L)	Fe (μg/L)	Mn (μg/L)	pH
0	0	34.3	14.7	2230	607	7.8
8	1	DL^a^	8.6	DL^a^	8.8	8.3
40	5	2.7	5.1	DL^a^	892	8.4
56	6.9	5.8	16.3	87	775	8.4
80	9.9	10.9	23.0	588	921	8.4
120	14.9	11.5	34.3	666	375	8.3
160	19.8	14.3	35.6	1090	415	8.4
240	29.7	8.9	17.4	233	652	NA^b^
280	34.7	7.7	15.3	164	598	8.5
296	36.6	13.3	30.2	690	406	8.5
315	39	12.3	21.5	518	494	8.5
r			0.904	0.933	− 0.725	
LOC			> 99%	> 99%	> 97.5%	

^a^DL means value was below the instrument detection limit

^b^NA means no measurement was taken

During these tests the flow was maintained at close to 0.125 mL/h and at the end the actual calculated flow rates averaged 0.1185 mL/h, 0.1238 mL/h and 0.1238 mL/h for the Mix1, Mix2 and Mix3 tests, respectively. Durations and treated volumes are shown in Tables 5, 6 and 7 to facilitate comparison with some of the earlier tests conducted.

**Fig. 3:**
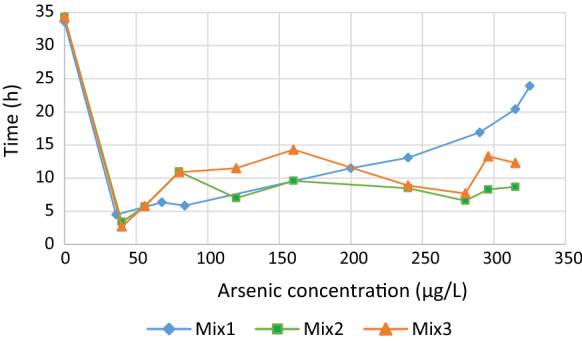
Comparison of effluent arsenic concentrations with time for the Mix1, Mix2 and Mix3 column tests

Effluent arsenic gradually increased with time for the Mix1 test while effluent arsenic concentrations fluctuated over time for the Mix2 and Mix3 tests and resulted in better overall arsenic removal (as illustrated in Fig. 3). Passive removal of arsenic from the groundwater from the town of Freshwater alone gave the smallest total arsenic removal, presumably due to the lower iron content, and Mix1 had the highest proportion of groundwater from the town of Freshwater and thus the lowest iron content. It could have been beneficial to extend the duration of the Mix2 and Mix3 tests as they did not stabilize, but time constraints prevented this.

### Comparison of 1:1, 1:3, Mix1, Mix2, and Mix3 test results

The 1:1 ratio of Wabana waters (Table 2) and the Mix1 water (Table 5) each had an initial arsenic concentration of 33.6 µg/L, though the iron concentration of the former of 5722 µg/L was almost ten times the iron concentration of the latter or 580 µg/L. To not surpass an effluent arsenic concentration of 7 µg/L only 4.0 L of the 1:1 ratio water but greater than 10 L of the Mix1 water could be treated. The Mix1 water had elements other than iron that were among the most significantly correlated with arsenic (Table 5) and one or more of them or some other factors could have assisted the arsenic removal. Alternatively, too much iron in water could end up inhibiting arsenic removal when oxidation and precipitation block the sand’s pores. Natural groundwater samples and their mixtures are chemically complex (Onorato et al. 2017) and although the water environment will determine how well the arsenic treatment system works (Sazakli et al. 2015), attributing causes and impacts could be difficult due to the complexity.

### Comparison of Mix1, Mix2, and Mix3 tests and tests with their makeup waters

Comparing tests with groundwater from the towns of Wabana and Freshwater together and separately and maintaining the effluent arsenic concentration at less than 7.0 µg/L, volumes of water that could be treated were 1.0 L of groundwater from the town of Freshwater, 1.4 L of Wabana groundwater (for which the initial arsenic concentration was roughly double that of the other waters), 10.0 L of Mix1 water, 6.9 L of Mix2 water and 6.9 L of Mix3 water. Adding some Wabana groundwater to the groundwater from the town of Freshwater greatly improved arsenic removal.

One possible reason for the improvement could have been that the extra iron in the Wabana groundwater compensated for the iron deficiency in the groundwater from the town of Freshwater. This is reasonable because among the three tests the Mix1 water had the least iron and the least total arsenic was removed.

The Wabana groundwater dilution with groundwater from the town of Freshwater could also have been a factor. Among the three tests, the best overall arsenic removal occurred for the Mix2 water with an Fe:As mass ratio of 32.2. In the Mix3 water, the Fe:As mass ratio was 65 and total arsenic removal was almost as good as for the Mix2 water. Thus, it again appears that too much iron could result in the filter becoming blocked sooner. Possibly the iron content was at the upper limit for ideal arsenic removal in the Mix3 water (2230 µg/L) under the specific test conditions encountered, such as the use of sand with a very small minimum grain size.

Other elements or factors could also have been important though their actual effects could be difficult to ascertain. Those elements correlated with arsenic in the Mix1, Mix2 and Mix3 tests changed as the water composition changed and this highlights the complexity of conducting tests on natural groundwater and their mixtures.

The strengths of this study are that natural groundwater was used in the testing and especially that tests were conducted on waters that were combinations of groundwater from the towns of Wabana and Freshwater. The authors have not seen another such study where groundwater from two locations was combined for arsenic removal and its greatly successful outcome is worth contemplating where the opportunity for a practical large scale application might arise, whether it be applied to groundwater or surface water.

A limitation of this study is that the Mix2 test in particular was not continued until effluent arsenic reached an equilibrium that was (very close to or) less than 10 µg/L and so it is not known the extent to which the Mix2 test might have been a special combination that contributed to its superior arsenic removal. One limitation in the analysis is related to the comparison of tests where water was allowed to flow by gravity through the sand column with tests where water was pumped through the column. The effect of pumping and the application of a stronger force could have meant that a greater depth of the sand media was used and this also contributed to the increased arsenic uptake but this potential effect could not be accounted for.

### Change in pH and its effect

The general increase in pH during Mix1, Mix2 and Mix3 column tests could have been due to a gradual oxygen depletion in the sand column over time as reducing environments are more basic than oxidizing environments. For example, sulfate reduction to sulfide consumes protons and so increases pH (Bohn et al. 1985).

The Ni, Mn and Zn were negatively correlated with arsenic. Increase in pH would increase removal of cationic divalent heavy metals and decrease removal of anionic arsenic, so this could explain the negative correlation. Arsenic release from sediments into water is very sensitive to change in pH (Shaw 2006) so even this small pH change could have led to arsenic release from the sand column.

Toward the end of the Mix2 test (Table 6) there is a reversal in the general pH trend and this test ends with the lowest pH among the three tests, as well as the smallest arsenic concentration in the effluent (Tables 5, 6 and 7 and Fig. 3).

### Elements correlated with arsenic in the Mix1, Mix2 and Mix3 tests

The elements for which significant correlations with effluent arsenic were found in the Mix1, Mix2 or Mix3 tests included Fe, Ni, Mn, Zn, Al, S, Cl and Br. There were some discrepancies between influent and effluent concentrations for Ni, Mn, Zn and Al, possibly due to fluctuations in concentrations between the two sampling times, though the trends in the effluent concentrations over time would not have been invalidated.

Effluent arsenic was correlated with effluent Cl, Br and S in the Mix1 test and effluent Al and Fe in the Mix3 test and these correlations occurred where the element concentrations among the three tests were the highest. Ni, Mn and Zi were also correlated with As but their relative abundances among the three tests did not appear to be a factor and instead as mentioned in the previous section they could have been responding to the change in pH as did arsenic but in the opposite direction.

Arsenic in sediments and groundwater in Vietnam was correlated with, in order of importance, Ni, Al and Mn (Berg et al. 2008) and arsenic released from mine tailings in the presence of humic acid has been correlated with Zn (Wang and Mulligan 2009). Ocinsaki et al. (2016) found that MnO_2_ could convert arsenite into arsenate and then adsorb the arsenate so both would be removed together but in the Mix1 and Mix3 tests as the effluent arsenic increased the Mn concentration decreased which suggests that the increasing pH was responsible for uptake of Mn. Anionic As, Cl and Br all increased in the effluent with increasing pH or increasing OH^−^ ions, with which they could have been competing.

Both ferric hydroxide and aluminum sulfate are able to take up arsenic (Rageh 2008) and this could explain why Al and S were positively correlated with arsenic. A correlation between Fe and As was seen only in the Mix3 test where Fe was the most abundant among the three similar tests.

## Conclusions

Iron deposition on the sand surface occurred when treating the high strength Wabana groundwater though scraping off the top layers of sand extended the life of the filter by providing a clean surface. SSFs could be an option for arsenic removal if treatment is supported in bench scale tests and if there are frequent removals of the top layer of sand, and SSFs are a simple and sustainable component of water treatment.

The greatest arsenic removal from the high strength Wabana groundwater occurred with active aeration of the sand media. Therefore, a better supply of oxygen to the sand media could be one factor in improving the arsenic removal by sand media. Since aeration with the vacuum pump compressed the sand and could also have interfered with arsenic removal, testing a sand with a greater minimum grain size and employing a selectively graded sand as in the design of RSFs might be considered for the purpose of further small scale testing.

Employing different means of supplying oxygen to the sand column helped illustrate the effect of oxygen in converting Fe^2+^ into Fe^3+^ which could in turn help promote conversion of As(III) into As(V) and encourage removal of the precipitated iron and arsenate by the sand media.

For water with suitable properties, a large scale SSF, possibly designed for gravity flow could be operated such that when the top layer of sand accumulates impurities, it is scraped off manually and washed and many layers can be removed before they are all replaced together. The energy output would be low and good quality treatment could be achieved. Such a design could virtually eliminate an environmental impact and using a RSF is also associated with a very low environmental impact.

Before the use of life cycles analysis, municipal structures were designed with the primary consideration being the initial capital costs, and long-term operation and maintenance costs were less accounted for even though they might end up representing a much greater percentage of the overall expense. If a more long-term approach is taken, then there may be many opportunities for redesigning water treatment facilities to better mitigate their environmental impact.

When the high strength Wabana groundwater and the treated Wabana water were combined, the arsenic and iron were diluted relative to the oxygen and this increased the volume of water that could be treated and the total arsenic that could be removed. This could be seen as a form of passive aeration and could have allowed more space within the media for natural oxidation of the ferrous iron and removal of arsenic. The findings of this study suggest that sand filtration could work better where arsenic concentrations are less than 35 µg/L, with better removals being possible the lower the arsenic concentrations. Lithium was correlated with arsenic removal in the 1:3 ratio water though the authors did not see any literature supporting its potential importance for arsenic removal.

Combining the high strength Wabana groundwater with groundwater from the town of Freshwater appeared to allow the high iron content of the former to compensate for the iron deficiency of the latter. Other studies have successfully tested the addition of iron filings and iron nails to sand filters to increase removal of arsenic and this study shows that adding an iron rich water is also effective.

Combining the high strength Wabana groundwater with groundwater from the town of the Freshwater diluted the arsenic and iron relative to the Wabana groundwater which could have allowed more space for removal of arsenic within the sand and possibly an ideal upper limit for the Fe:As mass ratio could be 65.

Other factors besides the presence of iron, its total concentration and its mass ratio with arsenic could also have been assisting the arsenic uptake. The variation in elements correlated with arsenic in the tests that combined groundwater from Wabana and groundwater from the town of Freshwater, showed variation depending on the composition of the water mixtures.

The correlations between arsenic and a number of other elements besides iron were encountered when Wabana groundwater was added to groundwater from the town of Freshwater but not when the high strength Wabana groundwater alone or mixtures of the two Wabana waters were tested. Arsenic removal was highest for the Mix1, Mix2 and Mix3 waters and lowest for the groundwater from the town of Freshwater alone, and so combing groundwater from the two different towns seemed to optimize conditions for arsenic removal.

Though other researchers have observed that Mn could be important for the removal of arsenic, this could not be demonstrated in this study. This could have been because arsenite was not abundant. Generally, the abundance of iron exceeded that of manganese by at least four times.

The pH increase during the Mix1, Mix2 and Mix3 tests could have been due to the increasingly reducing test conditions that enabled greater heavy metal uptake but caused arsenic to be released.

Overall, sand filtration could be a good option for arsenic removal, depending on the composition of the water to be treated. In light of the need to curb global energy consumption and transition to a more sustainable economy it could be worth investigating the use of SSFs for arsenic removal from drinking water on a larger scale. Purifying water by sand filtration has a long history and despite the larger land footprint compared to that required for conventional processes at modern treatment facilities, large scale SSFs have still been used. Some cities develop their water supplies by utilizing the closest water sources first, move further outward as the population expands, and eventually rely on multiple sources. At locations distant from cities land space may be more readily available and so siting of a SSF might not be precluded.
